# Supplementary material for: Genomic resistance in historical clinical isolates increased in frequency and mobility after the age of antibiotics
Source: bioRxiv. 2025 Jan 16:2025.01.16.633422. Preprint. [Version 1] doi: 10.1101/2025.01.16.633422 (PMC11761691; doi:10.1101/2025.01.16.633422)
Supplement: Supplement 2 [file NIHPP2025.01.16.633422v1-supplement-2.pdf]

bioRxiv preprint doi: https://doi.org/10.1101/2025.01.16.633422; this version posted January 16, 2025. The copyright holder for this preprint (which was not certified by peer review) is the author/funder, who has granted bioRxiv a license to display the preprint in perpetuity. It is made available under aCC-BY 4.0 International license.

Supplementary Figures

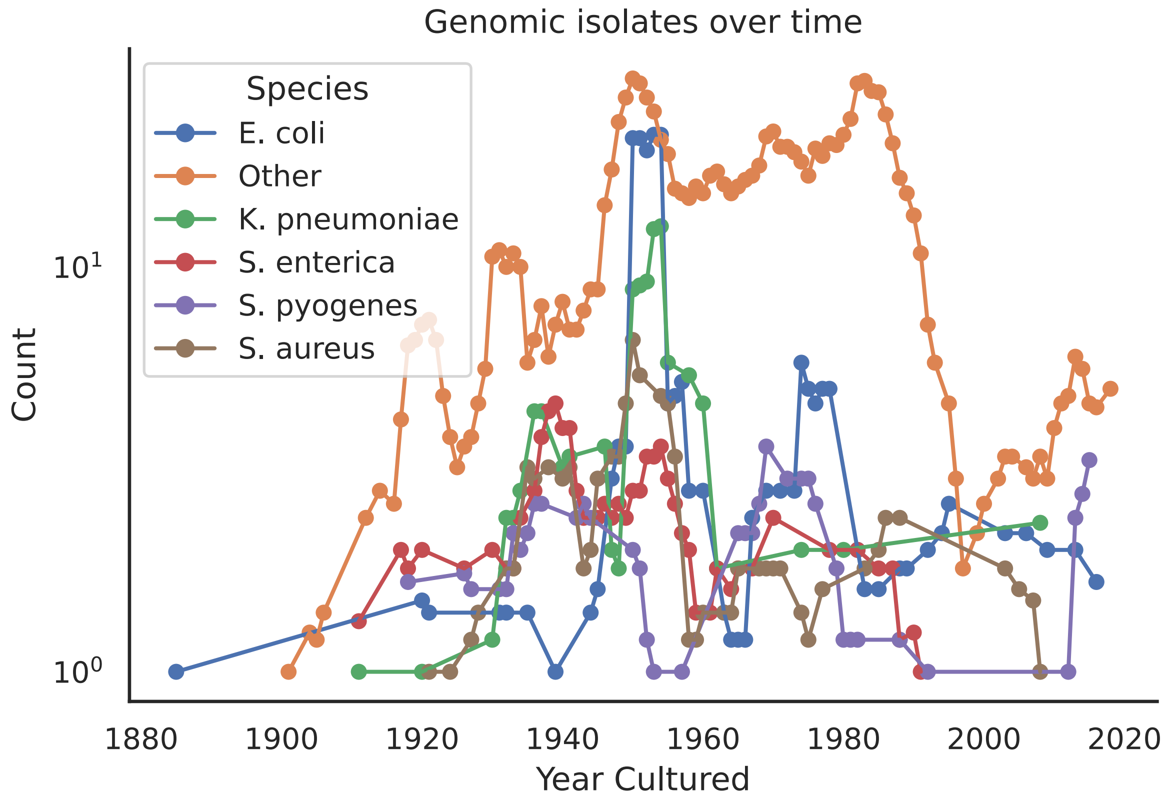

Supplementary Figure 1: Rolling count of isolates in the dataset over time. *Rolling average of number of isolates per specie in the data analyzed. Points represent the average number of isolates isolated within a window 5 years.*

bioRxiv preprint doi: https://doi.org/10.1101/2025.01.16.633422; this version posted January 16, 2025. The copyright holder for this preprint (which was not certified by peer review) is the author/funder, who has granted bioRxiv a license to display the preprint in perpetuity. It is made available under aCC-BY 4.0 International license.

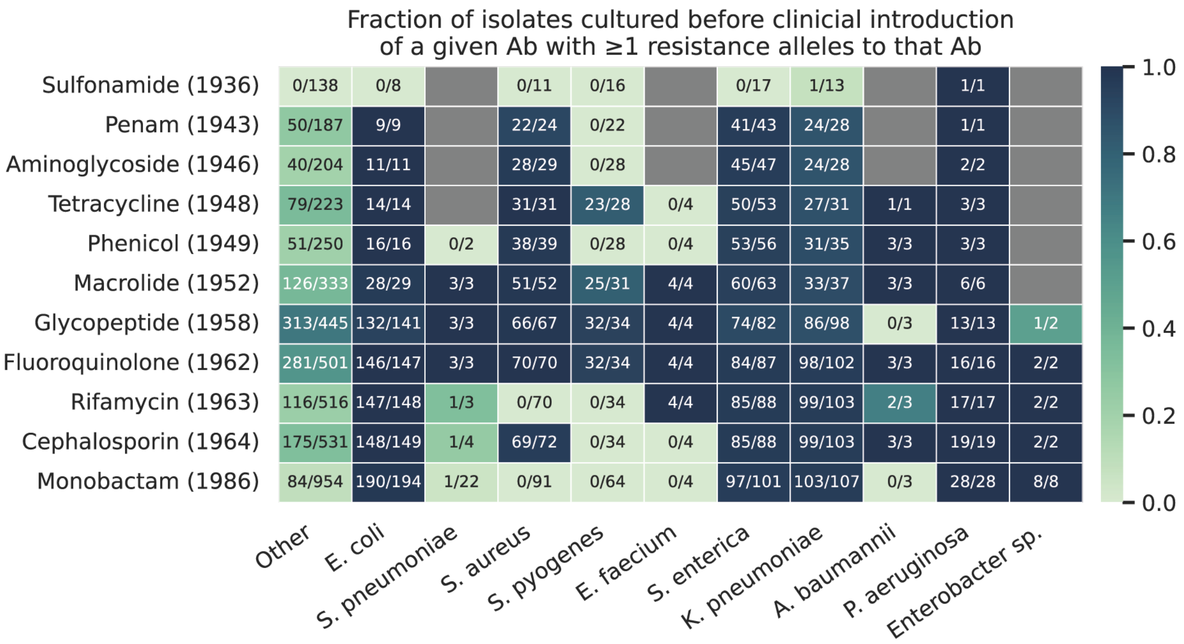

Supplementary Figure 2: Resistance-associated genomic elements known to exist within specific species' were also ubiquitous before clinical introduction of given antibiotics. *Heatmap representing the fraction of isolates harboring resistance alleles before the introduction of a given antibiotic. Denominator in each cell is the number of isolates of a given species cultured before the clinical introduction of the antibiotics in the corresponding row (represented by the (Year) next to the antibiotic). The numerator is the number of isolates cultured before clinical introduction of the antibiotic containing resistance alleles to that antibiotic. Resistance allele prediction and classification done from CARD without prior species knowledge.*

bioRxiv preprint doi: https://doi.org/10.1101/2025.01.16.633422; this version posted January 16, 2025. The copyright holder for this preprint (which was not certified by peer review) is the author/funder, who has granted bioRxiv a license to display the preprint in perpetuity. It is made available under aCC-BY 4.0 International license.

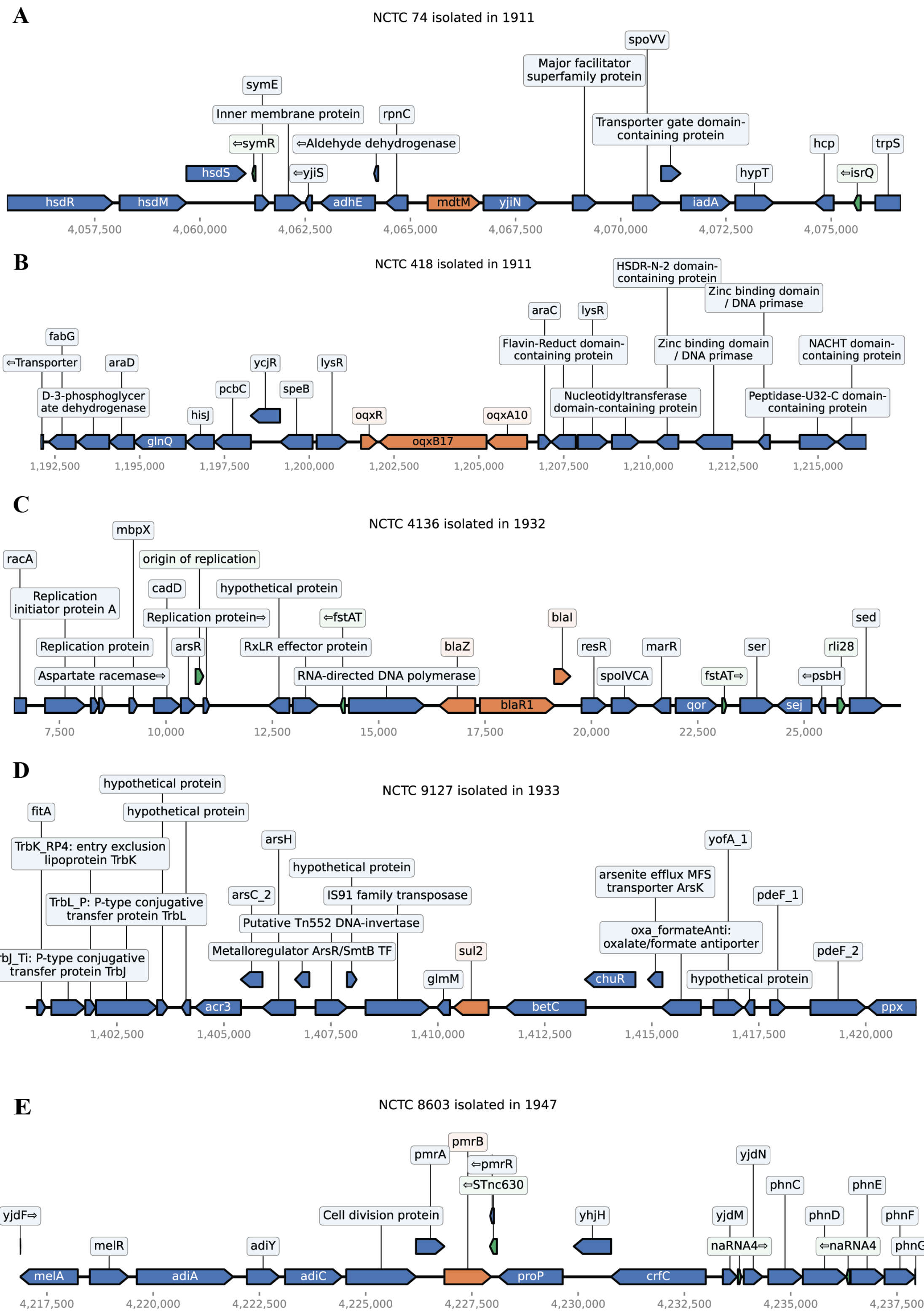

Supplementary Figure 3: Genomic neighborhood of pre-antibiotic era genomic resistance elements reveals similar genomic structures observed for these resistance alleles. *10kb genomic window surrounding each of the pre-antibiotic resistance elements in the isolates described in Box 1.*

bioRxiv preprint doi: https://doi.org/10.1101/2025.01.16.633422; this version posted January 16, 2025. The copyright holder for this preprint (which was not certified by peer review) is the author/funder, who has granted bioRxiv a license to display the preprint in perpetuity. It is made available under aCC-BY 4.0 International license.

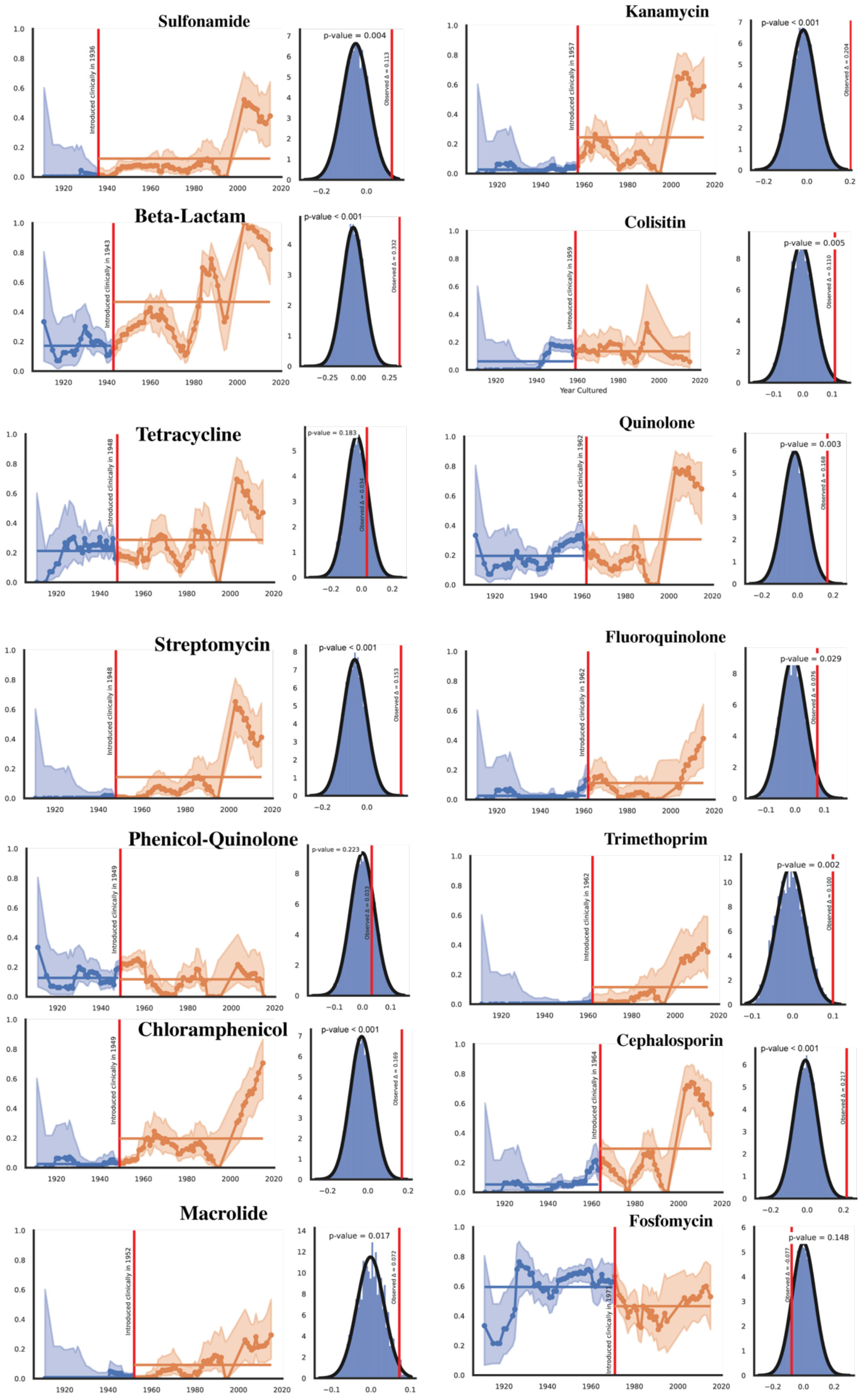

Supplementary Figure 4: Significant increase in observed resistance frequencies after clinical introduction of an antibiotic across most drug types. *See Figure 3 for plot explanation.*

bioRxiv preprint doi: https://doi.org/10.1101/2025.01.16.633422; this version posted January 16, 2025. The copyright holder for this preprint (which was not certified by peer review) is the author/funder, who has granted bioRxiv a license to display the preprint in perpetuity. It is made available under aCC-BY 4.0 International license.

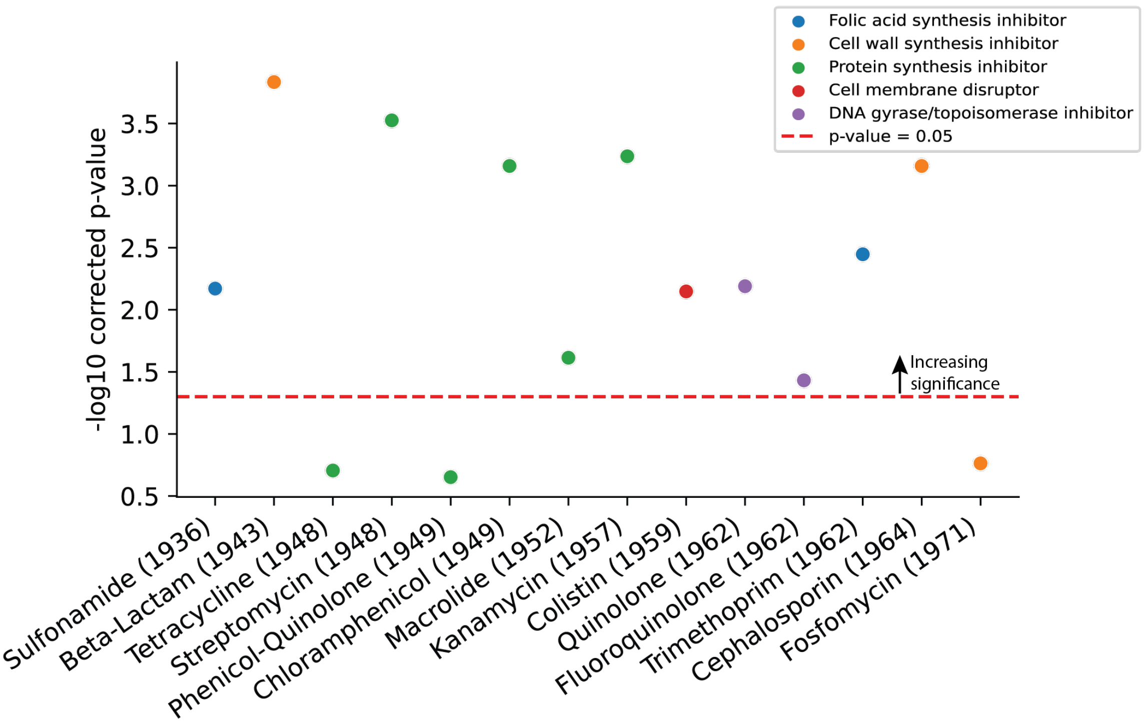

Supplementary Figure 5: Significant increase in observed resistance rates after clinical introduction are not associated with antibiotic mechanism. *Significance of change in resistance prevalence over all isolates and drug classes with 10,000 shuffles. Multiple hypotheses corrected with Benjamini-Hochberg. Individual points colored by antibiotic mechanism.*

bioRxiv preprint doi: https://doi.org/10.1101/2025.01.16.633422; this version posted January 16, 2025. The copyright holder for this preprint (which was not certified by peer review) is the author/funder, who has granted bioRxiv a license to display the preprint in perpetuity. It is made available under aCC-BY 4.0 International license.

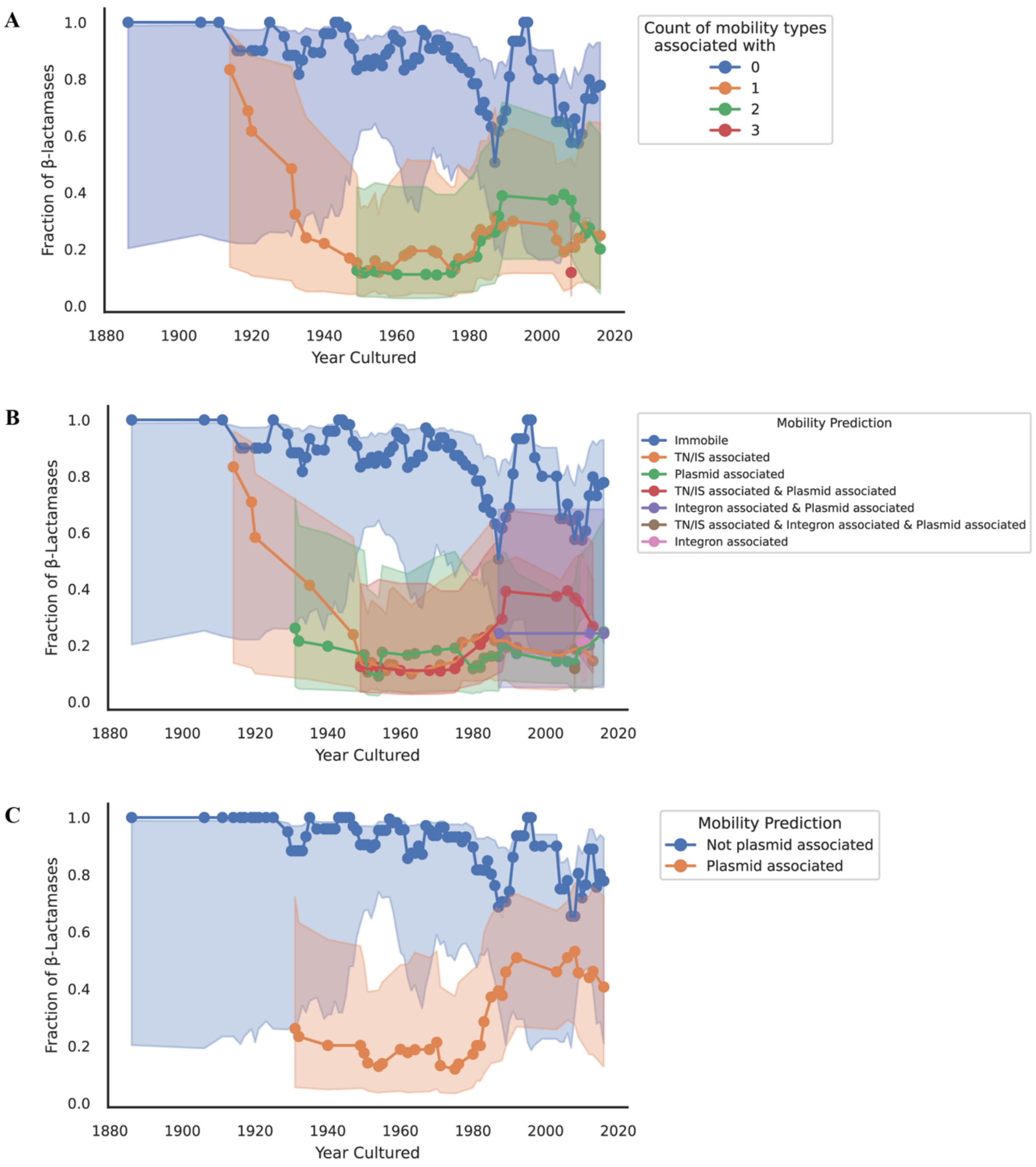

Supplementary Figure 6: Beta-lactamases experience increasing mobility over the years, with most mobility driven by plasmids. *Fraction of beta-lactamases classified as mobile over the course of the NCTC, shaded regions represent 95% confidence intervals based on sampling size (A) Number of mobility types associated with each beta-lactamase in the NCTC. (B) Breakdown of which combination of mobility types cause the counts in (A). (C) Fraction of beta-lactamases with some or no plasmid association.*

bioRxiv preprint doi: https://doi.org/10.1101/2025.01.16.633422; this version posted January 16, 2025. The copyright holder for this preprint (which was not certified by peer review) is the author/funder, who has granted bioRxiv a license to display the preprint in perpetuity. It is made available under aCC-BY 4.0 International license.

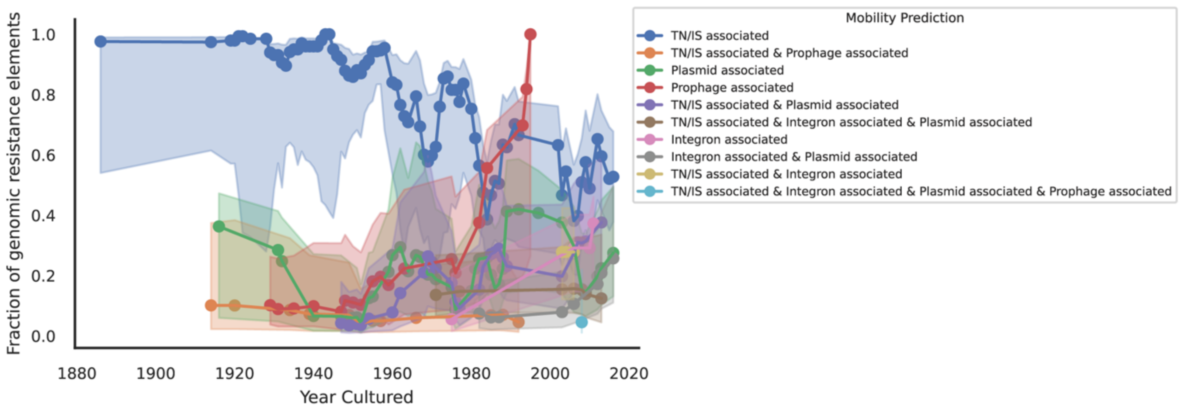

Supplementary Figure 7: Increase in mobility access of mobile resistance elements appears to be driven by an increase in Plasmid-associated resistance elements. *Fraction of mobile resistance elements as a function of time and the specific combination of mobility types observed. The shaded regions denote 95% confidence intervals from a beta distribution.*
